# Thermal Biology of *Liorhyssus hyalinus* (Hemiptera: Rhopalidae) and *Nysius simulans* (Hemiptera: Lygaeidae), Fed on the Milky Stage of Maize Grains

**DOI:** 10.1093/jisesa/ieac034

**Published:** 2022-07-03

**Authors:** Luis Cruces, Eduardo de la Peña, Patrick De Clercq

**Affiliations:** Department of Entomology, Faculty of Agronomy, Universidad Nacional Agraria La Molina, Lima 12, Peru; Department of Plants & Crops, Faculty of Bioscience Engineering, Ghent University, B-9000 Ghent, Belgium; Department of Plants & Crops, Faculty of Bioscience Engineering, Ghent University, B-9000 Ghent, Belgium; Instituto de Hortofruticultura Subtropical y Mediterránea “La Mayora (IHSM-UMA-CSIC), Spanish National Research Council 5CSIC, Estación Experimental “La Mayora”, Malaga, Spain; Department of Plants & Crops, Faculty of Bioscience Engineering, Ghent University, B-9000 Ghent, Belgium

**Keywords:** temperature, development, reproduction, hemipteran pests, quinoa

## Abstract

When quinoa, *Chenopodium quinoa* Willd., is cultivated in South America outside of its Andean origin, the heteropterans *Liorhyssus hyalinus* (Fabricius) and *Nysius simulans* Stål may emerge as important pests. Here we studied the development and reproduction of both species at different constant temperatures in the laboratory. Egg and nymphal development were investigated at 18, 22, 26, 30, 34, and 36°C. For both species, egg incubation time significantly decreased as the temperature increased. Nymphs did not successfully develop at 18°C and the total nymphal time significantly decreased as the temperature increased from 22 to 36°C. Based on a linear day-degree (DD) model, the lower developmental threshold (LDT) temperatures for eggs and nymphs were estimated to be 16.0 and 17.9°C for *L. hyalinus*, and 16.1 and 19.7°C for *N. simulans*, respectively. Thermal requirements for egg and nymphal development were 68.6 and 114.8 DD for *L. hyalinus*, and 77.7 and 190.3 DD for *N. simulans*, respectively. Reproduction and adult longevity were studied at 22, 26, 30, and 34°C. For both species preoviposition time decreased as temperature increased, and the oviposition period was longest at 26°C. The highest fecundity and egg viability were observed at 30°C, whereas longevities were higher at 22–26°C than at 30–34°C. As the lowest tested temperatures were not suitable to both heteropterans and 30°C was found to be the optimal temperature for development and reproduction, peak densities are expected in warm areas and seasons.

Quinoa (*Chenopodium quinoa* Willd.) is an Andean grain that has received increasing international attention due to its nutritional properties (Bedoya-Perales et al. 2018). Over the last decade, the crop area in South America where quinoa is cultivated, particularly in Bolivia and Peru, has been considerably expanded towards non-Andean regions at lower elevations (Cruces et al. 2016, Cruces et al. 2020a, Hinojosa et al. 2021). Moreover, quinoa has been cultivated for research and production around the world, in more than 120 countries (Alandia et al. 2020). 

When quinoa is cultivated outside of its Andean origin, it can be severely infested by a broader range of phytophagous insects (Cruces et al. 2020b). Two of these are the heteropteran pests *Liorhyssus hyalinus* (Fabricius) (Hemiptera: Rhopalidae) and *Nysius simulans* Stål (Hemiptera: Lygaeidae) which at the coastal level of Peru have been reported to cause serious problems in this crop (Cruces et al. 2016, Latorre 2017). Both species have also been noted to be part of the quinoa pest complex in Argentina and Chile (Dughetti 2015a, Chorbadjian et al. 2021).

*L. hyalinus* is a cosmopolitan species and in South America it has been reported from Argentina, Chile, Colombia, Ecuador, Paraguay, Peru, and Venezuela (Göllner-Scheiding 1976, Froeschner 1981, Cermeli et al. 2004, Hradil et al. 2007, Prado 2008, Chorbadjian et al. 2021). This bug has been recorded on a wide range of plants, both weeds and cultivated plants, and on the latter, it can become an important pest (Wheeler 2016). Adults of this rhopalid infest quinoa from the grain filling stage. Nymphs and adults suck water and nutrients from the developing grains, causing direct damage to quinoa production (Dughetti 2015a, Gómez and Aguilar 2016).

*N. simulans* is a neotropical species and has been noted to occur in Argentina, Brazil, Chile, Paraguay, Peru, and Uruguay (Dalazen et al. 2014, Cruces et al. 2020b, Chorbadjian et al. 2021). This soil dwelling species has a cryptic appearance and minute size and usually goes unnoticed by the farmer until its population grows and the insect starts climbing onto the weeds and crop plants. Like *L. hyalinus*, adults of *N. simulans* infest quinoa during the grain filling stage, and both the nymphs and adults suck on the developing grains, causing economic damage (Dughetti 2015a, 2015b; Gómez and Aguilar 2016; Cruces et al. 2020b).

The current study was undertaken to determine the effects of temperature on the developmental and reproductive parameters of *L. hyalinus* and *N. simulans* fed with fresh corn grains (*Zea mays* L.), an alternative host plant of these heteropterans (Dughetti 2015b, Wheeler 2016). The findings of the present study may be useful to predict their population dynamics in quinoa fields and make inferences on their potential distribution and peak densities throughout the year, according to the thermal conditions of the localities where quinoa is cultivated.

## Materials and Methods

### Stock Culture

Colonies of *L. hyalinus* and *N. simulans* were established in December 2018 with nymphs and adults collected in the quinoa fields of the Cereal and Native Grains Programme at the National Agrarian University La Molina, in Lima, Peru. Colonies of both species were established and maintained in the laboratories of the Museum of Entomology ‘Klaus Raven Büller’ at ambient laboratory conditions (around 26–28°C). The insects were housed in acrylic boxes of 20 × 20 × 20 cm with paper towelling on the bottom. The identity of *L. hyalinus* was confirmed with molecular tools: DNA extraction and PCR procedures were performed at the Department of Plants and Crops of Ghent University in Belgium (Cruces et al. 2021). *N. simulans* was identified by Dr. Pablo Dellapé from the Museo de La Plata in Argentina.

Adults and nymphs of both species were fed with fresh grains in the milky stage of amylaceous corn, which also served as a water source. For the adults of *N. simulans*, cotton rolls were provided as an oviposition substrate, where eggs were usually found individually or in small clusters of up to 10 eggs. For *L. hyalinus* no oviposition substrate was provided because eggs were laid on the corn grains and on the walls of the acrylic boxes, where they were usually found in clusters of around 10–20 eggs. Maintenance of the colony was done every 2–3 d during which all grains were replaced by fresh ones, dead individuals were removed and, for the containers with adults, eggs were collected to start a new generation.

## Experiments

Trials assessing developmental and reproductive parameters of both species were done in the laboratories of the Museum of Entomology ‘Klaus Raven Büller’, in a climatic cabinet (VISION SCIENTIFIC VS-3DM, South Korea) set at different constant temperatures (±0.5°C), 65 ± 5% relative humidity (RH), and a photoperiod of 14:10 (L:D) h.

For each species, adults from the stock culture were sexed, paired, and transferred (at least 100 pairs) to Petri dishes (9 cm diameter, 1.5 cm high, lined with white cardboard) to the corresponding temperature at which the developmental performance of the offspring was to be assessed.

On the third day, eggs (<1 h old) were collected to be used in the development assays: for *N. simulans*, the cotton (oviposition substrate) was examined under a binocular stereoscope in order to collect the eggs (that remained stuck to the cotton strands) with fine forceps; for *L. hyalinus*, the eggs were collected under a binocular stereoscope, aided with a piece of paper to separate the eggs from the surface to which they were attached. Only for the assay at 18°C, eggs (<1 h old) collected directly from the stock colony were used to determine the egg and nymphal development, since the females transferred to 18°C did not lay enough eggs for the experiment.

As in the stock culture, adults and nymphs in the different treatments were fed with fresh grains of corn which also served as a water source, and adults of *N. simulans* were provided with cotton rolls as oviposition substrate.

### Egg and Nymphal Development

Egg and nymphal development were studied at six constant temperatures: 18, 22, 26, 30, 34, and 36°C; except for the latter, these temperatures are in line with the yearly range of the daily maximum temperatures that may occur in the coastal areas of Peru (SENAMHI 2021). In all treatments, nymphs were fed with fresh grains of corn, which were replaced with fresh ones depending on the temperature, i.e., daily at 30–36°C or every other day at 18–26°C.

For each treatment, the incubation time of the eggs was determined using 100–230 eggs (<1 h old). To facilitate the counting of hatched eggs, and to prevent egg cannibalism by hatchlings in *N. simulans*, the eggs were stuck on the adhesive side of a piece of masking tape, placed on a plastic Petri dish, and kept at the studied temperature. As soon as the first egg hatched, the eggs were monitored every hour until the last egg hatched.

A second batch of eggs (<1 h old) was incubated at each temperature for monitoring nymphal development. From 80 to 140 first instars (1 day old) were individually caged in plastic Petri dishes (5 cm diameter, 1.3 cm high, lined with white cardboard) with a single fresh grain of corn. The nymphs were monitored at different time intervals according to the temperature, as follows: at the lowest temperatures (18 and 22°C), every 24 h; at mid-range temperatures (26 and 30°C) every 12 h, and at the highest temperature (34°C), every 8 h. At 36°C, a preliminary assay indicated that nymphs were very susceptible to manipulation, resulting in mortality of 98.5% in *L. hyalinus* and 81.4% in *N. simulans*. To increase the nymphal survival and enable determining the total nymphal period of a representative number of nymphs, instars were not monitored at the latter temperature, and nymphs were taken out of the incubator every 24 h only to replace the food.

Newly emerged adults (<12 h old) were sexed and weighed using a Mettler Toledo AL204 balance (Mettler-Toledo Group, China) and they were used in the assays to determine the reproductive parameters and longevity.

### Reproduction

Adult reproduction was studied at 22, 26, 30, and 34°C. Newly emerged adults (< 12 h old) coming from the nymphal development assays were paired and transferred to plastic Petri dishes (9 cm diameter, 1 cm high, lined with white cardboard) and then exposed to the same temperature and with the same food as in the nymphal period, but honeybee pollen was offered to the adults of *L. hyalinus* as a source of extra nutrients; preliminary observations indicated that *N. simulans* did not feed on the offered pollen. In all cases the minimum number of replicates (couples) was 11. Food (fresh corn and pollen grains) was replaced every other day.

Cotton rolls were provided as oviposition substrate to *N. simulans*, whereas the whole Petri dish could be used for oviposition by *L. hyalinus*. The cotton rolls or Petri dishes were checked daily until the first egg was laid to determine the preoviposition time. Thereafter, they were checked daily until the last egg was laid to calculate the oviposition period, but egg counts were done only every other day to determine total fecundity. Males were kept with their female mates until they died and longevity of both sexes was recorded.

In order to determine the egg viability (expressed by the percentage of egg hatching), all eggs laid by the monitored females at the different constant temperatures were stuck on the adhesive side of a piece of masking tape and placed on a plastic Petri dish, and then kept at the studied temperature until hatching.

## Data Analysis

All statistical analyses were performed using R software, version 4.0.5 (R Core Team 2020), and all tests were analyzed at the significance level α = 0.05.

For development and reproduction, differences between treatments were analyzed by using ANOVA tests, provided the data was normally distributed and homoscedastic as indicated by Shapiro Wilk and Bartlett tests, respectively. In case of heteroscedasticity, the Box–Cox transformation method was used to stabilize the variances; however, untransformed data are presented in the tables. Means were separated using a Tukey test. When data was not normally distributed, the nonparametric Kruskal–Wallis test was used to compare the treatments, followed by a Fisher’s least significant difference test as a post hoc test.

Parameters expressed as percentages (survival of nymphs, proportion of ovipositing females, and egg hatch) were compared by means of a logistic regression (family function = binomial) and groups were identified by the Tukey contrasts test. Means and SD-values were expressed as percentages. Calculations were performed in R, using the packages ‘*glm2*’ and ‘*multcomp*’ (Zhang and Rojas 2010, Marschner and Donoghoe 2018, Hothorn et al. 2022).

Sex ratios were evaluated versus an equal male:female distribution (1:1 ratio) using a nonparametric Chi-square test.

The linear relationship between temperature and development rate (*1/development time*) of eggs and nymphs was described by a linear day-degree model, which has been well documented to be suitable for estimation of lower development thresholds (LDTs) and thermal constants in several arthropods (Campbell et al. 1974, De Clercq and Degheele 1992, He et al. 2003, Du Plessis et al. 2011, Bonte et al. 2012, Luypaert et al. 2014, Mujica et al. 2017). The equation fitted was ‘*Y* = *a* + *bX*’, where *Y* is the development rate, *X* is the rearing temperature, and the regression parameters are the intercept (*a*), and the slope (*b*). The significance of the temperature in the fitted model was tested using a one-way ANOVA. The lower temperature thresholds of insect development were determined as the x-intercept (*t*_*o*_ = *−a/b*). For thermal requirements, the mean number of degree-days (DD) and standard deviations (from all individuals tested) were determined using the equation DD = *D*(*T−t*_*o*_) where *D* is the developmental time in days, *T* is the temperature (°C) during development, and *t*_*o*_ is the lower developmental threshold (°C) (De Clercq and Degheele 1992). LDTs and DD for the period from egg to preoviposition (as a single generation) were also calculated for both species. Thermal requirements of *L. hyalinus* and *N. simulans* were compared using a Mann–Whitney test.

## Results

### Development

All nymphal instar durations and the total nymphal period of both species varied significantly with temperature, decreasing as the temperature increased up to 34°C (for each monitored instar) or up to 36°C (for the total nymphal development) (Tables 1 and 2).

**Table 1.** Duration in days (mean ± SD) of the different instars and/or total nymphal period of *L. hyalinus* at six constant temperatures

Temp (°C)	Instar^*b*^						Total nymphal period^*b*^
	N^*a*^	N1	N2	N3	N4	N5	
18	140	10.02 ± 1.68a (43)	11.04 ± 1.81a (27)	11.92 ± 2.71a (25)	12.5 ± 2.35a (12)	/(0)	
22	125	5.52 ± 0.72b (115)	3.78 ± 0.43b (115)	3.40 ± 0.51b (115)	3.88 ± 0.53b (102)	6.25 ± 1.21a (70)	22.77 ± 2.04a (70)
26	80	4.23 ± 0.90c (70)	3.12 ± 1.00c (66)	3.18 ± 1.49c (60)	3.11 ± 0.59c (57)	4.90 ± 0.88b (51)	18.27 ± 1.97b (51)
30	112	2.16 ± 0.21d (102)	1.45 ± 0.10d (102)	1.47 ± 0.12d (85)	1.62 ± 0.12d (82)	2.67 ± 0.30c (78)	9.35 ± 0.38c (78)
34	96	1.28 ± 0.07e (92)	1.23 ± 0.18e (67)	1.21 ± 0.17e (44)	1.29 ± 0.14e (38)	2.28 ± 0.13d (36)	7.27 ± 0.39d (36)
36	110	/(83)	/(67)	/(56)	/(50)	/(55)	6.08 ± 0.51e (45)
X^2^		392.9	328.9	288.9	257.5	204.8	262.0
df		4	4	4	4	3	4

Different letters within a column indicate significant differences at α = 0.05 (Kruskal Wallis test).

At 36°C, instar periods (N1-N5) were not monitored.

^*a*^ Initial number of first instars tested.

^*b*^ The number of surviving nymphs based on which the mean and SD values were calculated is placed in parentheses.

**Table 2.** Duration in days (mean ± SD) of the different instars and/or total nymphal period of *N. simulans* at six constant temperatures

Temp (°C)	Instar^*b*^						Total nymphal period^*b*^
	N^*a*^	N1	N2	N3	N4	N5	
18	140	42.38 ± 6.71a (24)	/(0)	–	–	–	
22	82	16.93 ± 3.89b (70)	11.34 ± 2.45a (67)	9.41 ± 1.35a (64)	9.36 ± 0.99a (58)	12.25 ± 1.19a (52)	59.85 ± 7.13a (52)
26	98	9.37 ± 1.76c (94)	6.93 ± 1.38b (87)	6.35 ± 1.17b (83)	6.34 ± 0.94b (81)	8.01 ± 0.81b (79)	36.82 ± 4.03b (79)
30	88	4.72 ± 0.70d (86)	3.14 ± 0.47c (85)	2.97 ± 0.38c (83)	3.13 ± 0.41c (81)	4.53 ± 0.40c (80)	18.42 ± 1.59c (80)
34	101	2.36 ± 0.22e (99)	2.25 ± 0.29d (97)	2.10 ± 0.19d (97)	2.17 ± 0.23d (96)	3.44 ± 0.37d (93)	12.35 ± 0.78d (93)
36	87	/(76)	/(75)	/(75)	/(75)	/(73)	12.29 ± 0.96d (73)
X^2^		349.5	302.1	298.6	290.4	279.4	335.9
df		4	3	3	3	3	4

Different letters within a column indicate significant differences at α = 0.05 (Kruskal Wallis test).

At 36°C, instar periods (N1-N5) were not monitored.

^*a*^ Initial number of first instars tested.

^*b*^ The number of surviving nymphs based on which the mean and SD values were calculated is placed in parenthesis.

All instar durations could not be measured at the extreme temperatures of the tested range, i.e., at 18°C due to high mortality observed in the assay (Tables 1 and 2) and at 36°C where a high mortality in the preliminary assays was noted.

At 18°C, from the initial number of 140 first instars of *L. hyalinus* or *N. simulans*, not a single individual reached adulthood. For *L. hyalinus*, only 8.6% of the individuals reached the fifth instar, which eventually died within the following 12 d. For *N. simulans* only 17.1% of the individuals reached the second instar at 18°C, which eventually all died; the remaining 82.9% of the first instar nymphs progressively died within 10–66 d after hatching. At 22–34°C nymphs of both species successfully reached adulthood, but with an apparent higher mortality in *L. hyalinus* than in *N. simulans* (Tables 1 and 2).

The effects of temperature on nymphal survival (Table 3) were not compared statistically, because the nymphs were monitored at different time intervals in the different temperature treatments and therefore differences in the mortality rates among the treatments were probably also due to varying effects of manipulation. For instance, the nymphal survival of *L. hyalinus* at 36°C was slightly higher than at 34°C, probably because at the latter temperature the nymphs were examined (out of the climatic cabinet) every 8 h, whereas at 36°C the Petri dishes containing the nymphs were only taken out to replace the food every 24 h.

**Table 3.** Developmental parameters (mean ± SD) of *L. hyalinus* at six constant temperatures

Temp^*a*^ (°C)	Nymphal^*b*^ survival (%)	Egg incubation time (days)^*c*^	Nymphal period (days)		Adult weight (mg)		Sex ratio^*d*^ (male:female)
			Male	Female	Male	Female	
18	0.0 ± 0.0 (140)	25.38 ± 0.99a (107)	/	/	/	/	/
22	56.00 ± 4.40 (125)	11.54 ± 0.26b (148)	22.68 ± 1.51a	22.84 ± 2.39a	9.03 ± 0.90a	12.09 ± 1.35a	1:1.33
26	63.75 ± 5.37 (80)	7.86 ± 0.34c (156)	17.75 ± 2.12b	19.22 ± 2.32b	9.54 ± 1.10ab	12.27 ± 1.59a	1:0.82
30	69.64 ± 4.34 (112)	4.99 ± 0.06d (130)	9.23 ± 0.29c	9.45 ± 0.41c	8.80 ± 0.51b	11.44 ± 0.65b	1:1.23
34	37.50 ± 4.94 (96)	3.74 ± 0.06e (165)	7.03 ± 0.33d	7.49 ± 0.28d	8.04 ± 1.03c	9.64 ± 2.00c	1:1.12
36	40.90 ± 4.69 (110)	3.51 ± 0.04f (132)	5.99 ± 0.45e	6.18 ± 0.57e	7.97 ± 0.91c	10.26 ± 1.37c	1:1.05

Different letters within a column indicate significant differences at α = 0.05 (Kruskal Wallis test).

^*a*^ 69.3% of nymphs subjected to 18°C died in the first instar, 11.4% in the second instar, 1.4% in the third instar, 9.3% in the fourth instar, and 8.6% in the fifth instar.

^*b*^ The initial number of first instars tested is placed in parentheses.

^*c*^ The number of eggs tested is placed in parentheses.

^*d*^ Sex ratios did not differ significantly from a 1:1 ratio at α = 0.05 (*X*^*2*^ test).

Developmental times significantly varied with temperature for eggs (*L. hyalinus*: χ^2^= 816.83, df = 5, *P* < 0.001; *N. simulans*: χ^2^ = 890.44, df = 5, *P* < 0.001), male nymphs (*L. hyalinus*: χ^2^ = 124.39, df = 4, *P* < 0.001; *N. simulans*: χ^2^ =171.96, df = 4, *P* < 0.001), and female nymphs (*L. hyalinus*: χ^2^ =136.46, df = 4, *P* < 0.001; *N. simulans*: χ^2^ = 162.37, df = 4, *P* < 0.001), decreasing as the temperature increased from 18 to 36°C for eggs and from 22 to 36°C for nymphs (Tables 3 and 4).

**Table 4.** Developmental parameters (mean ± SD) of *N. simulans* at six constant temperatures

Temp^*a*^ (°C)	Nymphal^*b*^ survival (%)	Egg incubation time (days)^*c*^	Nymphal period (days)		Adult weight (mg)		Sex ratio^*d*^ (male:female)
			Male	Female	Male	Female	
18	0.0 ± 0.0 (140)	26.08 ± 0.92a (115)	/	/	/	/	/
22	63.41 ± 5.31 (82)	13.93 ± 0.26b (192)	58.92 ± 7.94a	60.70 ± 6.32a	1.64 ± 0.16a	2.39 ± 0.19a	1:1.08
26	80.61 ± 3.99 (98)	9.43 ± 0.49c (132)	36.77 ± 4.44b	36.93 ± 3.77b	1.49 ± 0.20b	2.39 ± 0.24a	1:0.75
30	90.91 ± 3.06 (88)	5.65 ± 0.12d (143)	18.36 ± 1.58c	18.44 ± 1.71c	1.56 ± 0.12a	2.41 ± 0.17a	1:0.86
34	92.08 ± 2.69 (101)	4.23 ± 0.15e (229)	12.36 ± 0.77d	12.34 ± 0.79d	1.40 ± 0.13c	2.33 ± 0.18a	1:1.11
36	83.91 ± 3.94 (87)	4.06 ± 0.13f (132)	12.32 ± 0.96d	12.26 ± 0.97d	1.22 ± 0.11d	2.02 ± 0.13b	1:1.15

Different letters within a column indicate significant differences at α = 0.05 (Kruskal Wallis test).

^*a*^ 87.9% of nymphs subjected to 18°C did not reach the second instar, the remaining 12.1% died in the second instar.

^*b*^ The initial number of first instars tested is placed in parentheses.

^*c*^ The number of eggs tested is placed in parentheses.

^*d*^ Sex ratios did not differ significantly from a 1:1 ratio at α = 0.05 (*X*^*2*^ test).

The heaviest males and females of *L. hyalinus* emerged at 22 and 26°C, whereas the lightest were observed at 34 and 36°C (males: χ^2^ = 50.67, df = 4, *P* < 0.001; females: χ^2^ = 53.26, df = 4, *P* < 0.001) (Table 3). For *N. simulans* the heaviest males were observed at 22 and 30°C, but females had similar weights at 22–34°C; the lightest males and females emerged at the extreme temperature of 36°C (males: χ^2^ = 91.51, df = 4, *P* < 0.001; females: χ^2^ = 73.19, df = 4, *P* < 0.001).

No significant deviations from a 1:1 ratio were found in the sex ratio of *L. hyalinus* at 22°C (χ^2^ = 1.43, *P* = 0.232), 26°C (χ^2^ = 0.49, *P* = 0.484), 30°C (χ^2^ = 0.82, *P* = 0.365), 34°C (χ^2^ = 0.11, *P* = 0.739), and 36°C (χ^2^ = 0.02, *P* = 0.881). Likewise, proportions of males and females of *N. simulans* were similar at 22°C (χ^2^ = 0.08, *P* = 0.781), 26°C (χ^2^ = 2.27, *P* = 0.132), 30°C (χ^2^ = 0.50, *P* = 0.479), 34°C (χ^2^ = 0.27, *P* = 0.604), and 36°C (χ^2^ = 0.34, *P* = 0.558).

### Day-degree Model

**Fig. 1.**
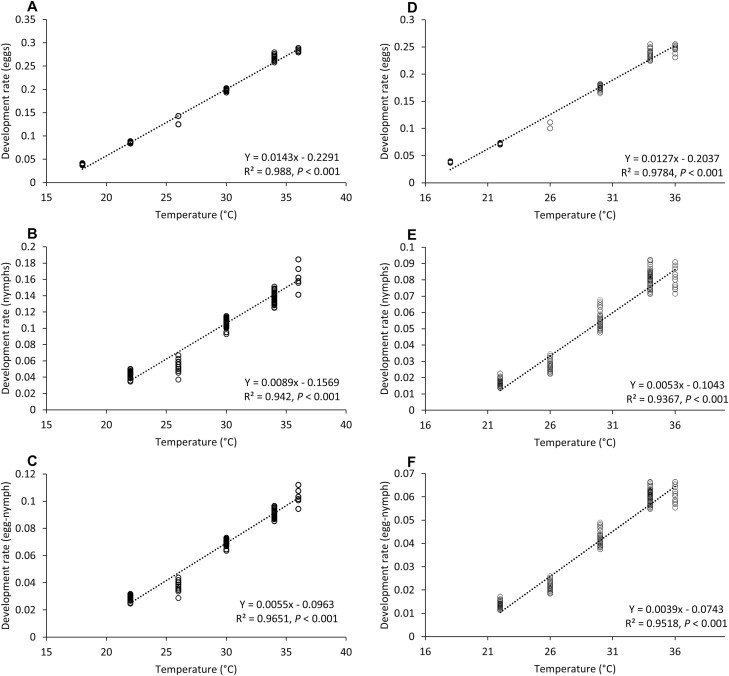
Linear relationship between temperature and developmental rate of egg, nymphal, and egg–nymphal stages of *L. hyalinus* (A, B, and C) and *N. simulans* (D, E, and F). Broken lines represent linear regressions of all data from 18°C (eggs) or 22°C (nymphs) to 36°C.

The linear regression analysis of the relationship between temperature and development rate of the egg and nymphal stage indicated a good linear model fit both for *L. hyalinus* and *N. simulans* at the range of temperatures from 18°C (eggs) or 22°C (nymph and egg–nymph periods) to 36°C (in all cases *R*^2^ > 93% and *P* < 0.001) (Fig. 1; Table 5). The egg and nymphal development of *L. hyalinus* required 68.6 and 114.8 DD, respectively, and an LDT of 16.0°C for eggs and 17.9°C for nymphs was estimated. For *N. simulans*, eggs and nymphs required 77.7 and 190.3 DD to complete development, respectively; the LDT for eggs was 16.1°C while for nymphs it was 19.7°C. The thermal requirements and lower threshold temperature for one generation (egg-preoviposition) were 236.9 DD and 18.0°C for *L. hyalinus* and 301.5 DD and 19.0°C for *N. simulans.*

**Table 5.** Lower developmental thresholds (t_o_), degree-day requirements (K) (means ± SD), and linear regression equations with corresponding coefficients of determination *(R*^*2*^) for the immature stages and for the egg-preoviposition period of *L. hyalinus* and *N. simulans* calculated for constant temperatures from 18°C (eggs) or 22°C (nymphs) to 36°C

Species	Stage	t_o_ (°C)	K(DD)	Regression equation	*R* ^*2*^	*F-*value	*P*-value
*L. hyalinus*	Egg	16.0	68.6 ± 8.0	Y = −0.2291 + 0.0143X	0.988	68350	<0.001
	Nymph	17.9	114.8 ± 20.2	Y = − 0.1569 + 0.0089X	0.942	4500	<0.001
	Egg–Nymph	17.5	181.7 ± 24.2	Y = − 0.0963 + 0.0055X	0.965	7686	<0.001
	Egg-Preoviposition	18.0	236.9 ± 38.6	Y = − 0.0759 + 0.0042X	0.956	3174	<0.001
*N. simulans*	Egg	16.1	77.7 ± 11.93	Y = − 0.2037 + 0.0127X	0.9784	42560	<0.001
	Nymph	19.7	190.3 ± 33.4	Y = − 0.1043 + 0.0053X	0.937	5549	<0.001
	Egg–Nymph	19.3	260.5 ± 38.4	Y = − 0.0743 + 0.0039X	0.952	7413	<0.001
	Egg-Preoviposition	19.0	301.5 ± 39.1	Y = − 0.0634 + 0.0033X	0.958	4244	<0.001

Degree Day requirements for development of the egg and nymphal stages were significantly higher in *N. simulans* than in *L. hyalinus* (*P* < 0.001).

### Reproduction and Longevity

No differences were found in the proportion of ovipositing females at 22, 26, 30, and 34°C both for *L. hyalinus* (χ^2^ = 3.98, df = 3, *P* = 0.263) and *N. simulans* (χ^2^ = 1.31, df = 3, *P* = 0.727) (Tables 6 and 7).

**Table 6.** Reproductive parameters and longevities (means ± SD) of *L. hyalinus* at four constant temperatures

Temp (°C)	Proportion of ovipositing females^*a*^ (%)	Preoviposition period (days)	Oviposition period (days)	Fecundity^*b*^ (eggs/female)	Egg Hatch (%)^*b*^	Longevity (days)	
						Male^*c*^	Female^*c*^
22	88.9 ± 0.1a (18)	13.8 ± 4.0a	56.9 ± 28.8a	169.5 ± 119.3c	30.1 ± 0.9c	94.5 ± 27.7a	79.3 ± 19.9a
26	100.0 ± 0.0a (11)	11.8 ± 3.1a	68.2 ± 31.2a	275.4 ± 121.6bc	59.7 ± 0.9b	88.9 ± 26.1a	85.2 ± 25.1a
30	100.0 ± 0.0a (17)	6.3 ± 2.5b	44.0 ± 9.9a	552.8 ± 158.3a	67.1 ± 0.5a	53.8 ± 19.4b	51.7 ± 13.8b
34	93.8 ± 0.1a (16)	3.6 ± 1.1c	29.9 ± 9.4b	384.7 ± 132.7b	66.6 ± 0.6a	43.3 ± 10.8b	39.9 ± 8.7b

Different letters within a column indicate significant differences at α = 0.05: Tukey contrast test (ovipositing females and egg hatch), Kruskal Wallis test (preoviposition and oviposition period), Tukey test (fecundity, adult longevity).

^*a*^ The number of adult pairs tested at each temperature is placed in parentheses.

^*b*^ Based on the total number of eggs laid per treatment.

^*c*^ ANOVA run after using Box–Cox transformation, λ = 0.5.

**Table 7.** Reproductive parameters and longevities (means ± SD) of *N. simulans* at four constant temperatures

Temp (°C)	Proportion of ovipositing females^*a*^(%)	Preoviposition period (days)	Oviposition period^*b*^ (days)	Fecundity (eggs/female)	Egg hatch (%)^*c*^	Longevity (days)	
						Male^*d*^	Female^*e*^
22	95.7 ± 4.3a (23)	8.3 ± 1.6a	29.1 ± 8.6b	94.6 ± 56.6c	15.85 ± 0.8c	58.9 ± 15.9a	45.2 ± 11.5b
26	93.3 ± 6.4a (15)	5.3 ± 1.0b	51.9 ± 15.8a	265.6 ± 95.1ab	62.59 ± 0.8b	63.9 ± 25.0a	58.3 ± 15.9a
30	95.7 ± 4.3a (23)	2.8 ± 0.5c	27.6 ± 10.1b	299.5 ± 79.2a	95.91 ± 0.2a	34.9 ± 10.6b	34.7 ± 11.9c
34	100.0 ± 0.0a (13)	2.4 ± 0.2d	27.9 ± 8.1b	200.8 ± 59.2b	62.18 ± 0.9b	34.6 ± 10.7b	33.9 ± 6.7c

Different letters within a column indicate significant differences at α = 0.05: Tukey contrast test (ovipositing females and egg hatch), Kruskal Wallis test (preoviposition period), Tukey test (oviposition period, fecundity, adult longevity).

^*a*^ The number of adult pairs tested at each temperature is placed in parentheses.

^*b*^ ANOVA run after using Box–Cox transformation, λ = 0.1.

^*c*^ Based on the total number of eggs laid per treatment.

^*d*^ ANOVA run after using Box–Cox transformation, λ = − 0.3.

^*e*^ ANOVA run after using Box–Cox transformation, λ = − 0.1.

For *L. hyalinus* the preoviposition periods ranged from 3.6 to 13.8 d, and they significantly decreased as temperature increased, although there were no differences at the lower temperatures of 22 and 26°C (χ^2^ = 43.14, df = 3, *P* < 0.001). The oviposition periods ranged from 29.9 to 56.9 d, and were similar at 22, 26, and 30°C, but significantly lower at 34°C (χ^2^ = 19.99, df = 3, *P* < 0.001) (Table 6).

Preoviposition times for *N. simulans* varied from 2.4 to 8.3 d, and they significantly decreased with increasing temperature (χ^2^ = 61.03, df = 3, *P* < 0.001). The oviposition period was highest at 26°C with 51.9 days on average, but there were no differences at 22, 30, and 34°C (*F* = 14.1, df = 3, *P* < 0.001) (Table 7).

The lowest temperature (22°C) significantly affected the fecundity both for *L. hyalinus* (*F* = 23.6, df = 3, *P* < 0.001) and *N. simulans* (*F* = 31.9, df = 3, *P* < 0.001). The highest fecundity was obtained at 30°C with 553 eggs/female on average for *L. hyalinus* and 300 eggs for *N. simulans*; in the latter species, there were no differences in fecundity at 26 and 30°C (Tables 6 and 7).

**Fig. 2.**
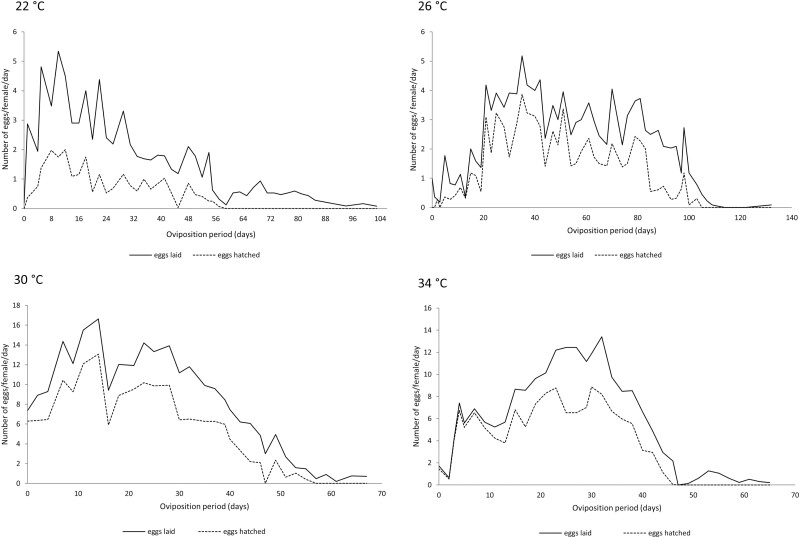
Daily mean fecundity and daily mean egg viability of *L. hyalinus* during its oviposition period at 22, 26, 30, and 34°C.

**Fig. 3.**
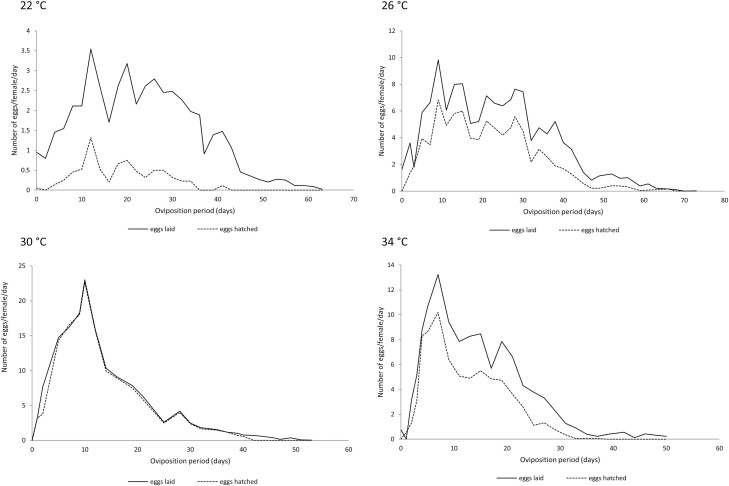
Daily mean fecundity and daily mean egg viability of *N. simulans* during its oviposition period at 22, 26, 30, and 34°C.

Egg hatch during the total oviposition period was significantly affected by temperature regime (Figs. 2 and 3). Egg hatch for both *L. hyalinus* (χ^2^ = 1289.7, df = 3, *P* < 0.001) and *N. simulans* (χ^2^ = 5705.7, df = 3, *P* < 0.001) was lowest at 22°C: 30.1% and 15.9%, respectively. The highest egg hatch was observed at 30°C: 95.9% for *N. simulans* and 67.1% for *L. hyalinus* (Tables 6 and 7). For both heteropterans, the lowest tested temperature (22°C) negatively affected the daily fecundity and egg hatch rates, while at 30°C these rates reached higher values as compared with the other temperatures (Figs. 2 and 3).

Adult longevity of *L. hyalinus* decreased as the temperature increased, being the longest at 22 and 26°C and shortest at 30 and 34°C, both for paired males (*F* = 21.76, df = 3, *P* < 0.001) ranging from 43.3 (34°C) to 94.5 (22°C) d on average, and for paired females (*F* = 24.30, df = 3, *P* < 0.001) ranging from 39.9 (34°C) to 85.2 (26°C) d on average (Table 6). The longest longevity of paired males of *N. simulans* was observed at 22 and 26°C and the shortest at 30 and 34°C (*F* = 18.9, df = 3, *P* < 0.001) ranging from 34.6 (34°C) to 63.9 (26°C) d on average; for paired females, the longest longevity was recorded at 26°C and the shortest at 30 and 34°C (*F* = 13.7, df = 3, *P* < 0.001), ranging from 33.9 (34°C) to 58.3 (26°C) d on average (Table 7).

## Discussion

Few studies have addressed the biology of *L. hyalinus* and life history data provided in the literature are scattered, poorly described, or are in the grey literature (Readio 1928, Hradil et al. 2007, Cornelis et al. 2012, Ríos 2014, Arenas 2019). Similarly, little is known on the biology of *N. simulans* and much of the work is unpublished (Mamani 2015, Vásquez 2016, Maquera 2018). In this context, the present study provides more detailed information on the developmental and reproductive biology of both hemipteran pests.

Developmental rates of the eggs and nymphal instars of *L. hyalinus* and *N. simulans* increased significantly with temperature from 18°C (eggs) or 22°C (nymphs) to 36°C. At the lowest tested temperature (18°C) there was a pronounced prolongation of the egg incubation time, but the nymphs that emerged at this temperature did not reach adulthood; however, the fact that 8.6% of the nymphs of *L. hyalinus* reached the fifth instar and that 82.9% of the *N. simulans* first instars stayed alive within a range of 10–66 d after hatching, may suggest that the nymphs can tolerate 18°C for some period of time. Since eggs used in the assay at 18°C were those that had been laid by females from the stock colony at a higher temperature (26–28°C), these results can be partly influenced by maternal effects (Mousseau and Dingle 1991, Gilchrist and Huey 2001).

Results from previous studies in grey literature on the development of *L. hyalinus* and *N. simulans* are difficult to compare with our findings because they were carried out under ambient laboratory conditions, with varying temperatures and relative humidity yielding nonreplicable results (Mamani 2015, Vásquez 2016, Maquera 2018, Arenas 2019). Ríos (2014) studied the biology of *L. hyalinus* fed with quinoa at 21.8 ± 1.3°C and 48.3 ± 8.3% RH. The latter author registered mean egg incubation and nymphal periods of 13.95 and 28.11 d, similar to our findings at 22°C. Atalay (1978, cited in Hradil et al. (2007)) reported that eggs hatched after 5 d at 25°C and after 3 d at 34°C; the development of the first fours instars (N1–N4) took on average 2 days each at 25°C and 1 day at 34°C, whereas for N5 it took 3 days at 25°C and 2 days at 34°C; these results differ substantially from our findings.

Sex ratios both in *L. hyalinus* and *N. simulans* were essentially 1:1 at the different constant temperatures, suggesting that males or females do not have a selective survival advantage as a function of temperature. Similar ratios were observed in previous studies carried out at temperatures within the range of the current study, for *L. hyalinus* (Ríos 2014, Arenas 2019) and *N. simulans* (Hradil et al. 2007, Mamani 2015, Vásquez 2016, Maquera 2018). A sex ratio of 1:1 was also found in a related species, *Nysius huttoni* White, at different constant temperatures (He et al. 2003).

The thermal requirements for immature development and the estimated lower thresholds for the nymphal development of *N. simulans* (260.5 DD and 19.3°C) were higher than those of *L. hyalinus* (181.7 DD and 17.5°C). This suggests that *L. hyalinus* may develop at lower temperatures than *N. simulans* and may complete more generations through the year. Such LDTs are relatively higher than those reported for other species, and only in few species the LDTs were found between 17 and 20°C (e.g. *Liposcelis paeta* Pearman (Psocoptera: Liposcelididae), *Tribolium castaneum* (Herbst) (Coleoptera: Tenebrionidae), and *Latheticus oryzae* Waterhouse (Coleoptera: Tenebrionidae)) (Stejskal et al. 2019). However, there are various factors that may affect the LDTs, including the geographical distribution of the species and specific thermal adaptations of local strains, which may result in substantial intraspecific variation in LDT values (Stejskal et al. 2019).

The lower developmental threshold calculated for *L. hyalinus* (17.5°C) is in line with the findings of Atalay (1978), but this author mentioned a thermal constant (218.4 DD) much higher than that found in the present study (181.7 DD) (Table 8). There has been no other previous attempt to determine the thermal requirements and lower thresholds for *L. hyalinus* nor for *N. simulans*.

**Table 8.** Lower developmental thresholds (t_o_), degree-day requirements (K), and maximum fecundities of *N. ericae*, *N. huttoni*, *N. vinitor*, and *L. hyalinus* reported in the literature

Species	Egg		Nymph		Egg–Nymph		Fecundity^*a*^	Source
	to(°C)	K(DD)	to(°C)	K(DD)	to(°C)	K K(DD)		
*N. ericae*	13.9	74.6	15.2	200	14.8	270.3	246 (26°C), 276 (28°C)	Du Plessis et al. 2011
*N. huttoni*	11.5	96.9	10	588	11.8	625	12.93 (20°C), 11.0 (25°C)	He et al. 2003
*N. vinitor*	14.5	77	15	225	n.a.	n.a.	578 (25°C), 542 (°C)	Kehat and Wyndham, 1972
*L. hyalinus*	n.a.	n.a.	n.a.	n.a.	n.a.	n.a.	558 (ca 30°C)	Readio 1928
	n.a.	n.a.	n.a.	n.a.	17.2	218.4	n.a.	Atalay (1978)

n.a. not available.

^*a*^ Temperatures at which the maximum fecundities were observed are placed in parentheses.

The thermal biology has been documented for other *Nysius* species that are agricultural pests, including *N. huttoni*, *Nysius vinitor* Bergroth, and *Nysius ericae* Schilling (Slater 1964, Kehat and Wyndham 1972, He et al. 2003, Du Plessis et al. 2011). In all of these studies, the *Nysius* spp. were fed on sunflower seeds. The estimated low thresholds for development reported for *N. huttoni*, *N. vinitor*, and *N. ericae* are substantially lower than our results (Table 8). However, the degree-day requirements calculated in the current study are similar to those obtained for *N. ericae* and *N. vinitor* but very different from those in *N. huttoni* (Table 8). These differences may be in part attributed to the geographical distribution of the (sub)tropical vs temperate species: *N. huttoni* is native to New Zealand with a mean annual temperature ranging from 10 to 16°C (Mullan et al. 2006).

Temperature did not have a significant effect on the proportion of ovipositing females, but it did have on the other reproductive parameters. Lower temperatures had a negative impact on oviposition: when females of either species from the stock colony (26–28°C) were transferred to an incubator at 18°C to collect their eggs, the oviposition rate decreased; in both species, the lowest fecundity and egg hatch were observed at 22°C. A trend towards lower fecundity of *L. hyalinus* as temperature decreased was also recorded by Atalay (1978). In the present study, the most optimal temperature for both species was 30°C, at which the highest fecundity and egg viability were observed. The maximum fecundity of *L. hyalinus* was on average 553 eggs/female, similar to the observations of Readio (1928) in summer conditions with temperatures at midday above 30°C (Table 8). The maximum fecundity of *N. simulans* at 26 and 30°C averaged 266 and 300 eggs/female, respectively, which is in line with the values reported for *N. ericae*, but considerably lower than those reported for *N. vinitor*. For *N. huttoni*, a very low fecundity was registered (Table 8).

Our results indicate that both heteropterans are not well adapted to the temperatures of 18 and 22°C: the lowest fecundity was observed at 22°C and at 18°C the nymphs could not reach adulthood. This may explain why both species have not been recorded in the highlands of Peru characterized by its relatively low temperatures;e.g., in Jauja (over 3,000 m a.s.l.) the annual maximum temperature averages 19.5 ± 2.2°C, whereas the minimum temperature averages 4.5 ± 3.4°C (data from 2008 to 2012, SENAMHI 2021)). At lower elevations (i.e., Majes, 1, 410 m a.s.l.; La Molina, 343 m a.s.l.; Olmos, 175 m a.s.l.), however, where these heteropterans have been recorded causing damage on crops, the annual maximum temperatures are around 25°C (data from 2009 to 2013, SENAMHI 2021)), which constitute better conditions for these species (Cruces et al. 2016, Gómez and Aguilar 2016, Latorre 2017, Cruces et al. 2020b, Soca 2021).

Data of reproductive parameters suggests that 30°C is optimal for both species; this temperature is usually reached in summer (January to March) in the coastal region (SENAMHI 2021). Considering that quinoa in the coastal region of Peru is usually sown in winter (i.e., between June to August), in late sowings the crop maturation and harvest coincide with the summer (in January) when peak numbers of *L. hyalinus* and *N. simulans* are present (Gómez and Aguilar 2016). Recent studies have yielded suitable varieties of quinoa adapted to warm conditions for spring–summer sowings; however, the promising varieties will eventually be faced with the phytosanitary problems posed by these bugs when cultivated on a larger scale (Villena 2011, Marca 2015, Antezana-Febres et al. 2019).

Taking as a reference the meteorological data of Lima, where the daily mean temperature during the year ranges approximately from 17.5 to 25.5°C (averaging 21.5°C), and based on the DD requirements for egg to preoviposition (236.9 DD), an average of 5.3 generations of *L. hyalinus* and 3.0 generations of *N. simulans* can theoretically be expected in a year, meaning that both species are multivoltine.

The current study contributes to a better understanding of the geographical distribution of *L. hyalinus* and *N. simulans* in Peru based on the temperature regimes characterizing the regions where quinoa has been cultivated. The information gathered may be useful from an agronomic point of view to improve the management of quinoa. For instance, it may assist in settling proper sowing times of quinoa, avoiding late sowings in order to prevent the coincidence of grain maturation with periods in the summer when peak pest numbers are expected (Gómez and Aguilar 2016). Including a fallow period or practicing a crop rotation system during the summer may be a key strategy to prevent damage by both heteropteran pests. Our results may be also useful as a starting point to lead further studies determining the lethal temperatures of both heteropterans, their survival mechanisms at unfavourable temperatures, and their performance on other diets.
